# The Conservative Treatment of Teeth by Gold Process

**Published:** 1893-07

**Authors:** Francis Peabody

**Affiliations:** Louisville, Ky.


ARTICLE IV.

### THE CONSERVATIVE TREATMENT OF TEETH BY GOLD PROCESS.

BY FRANCIS PEABODY, LOUISVILLE, KY.

Read before the Mississippi State Dental Society, April 6th, 1893.

The conservation of the dental organs has been, from the commencement of the art of dentistry up to the time when that calling was recognized as a profession, the first aim of those who had the true welfare of humanity at heart.

In the earlier days, when but little was known of the dental organism, efforts were made in a crude way, with crude materials, (lead being used, perhaps, more than any other article,) to save the teeth from destruction by decay, and, to a certain extent, success crowned the efforts of those engaged in this work. Little by little a better understanding of the requirements of stoppings was attained, advancements were made with filling materials, until the royal virgin metal gold came to be recognized as the one thing by which the best results were to be obtained, and in our present state of attainments this metal still holds the supremacy. Are we correct in this? Are we justified in maintaining that gold is the best material to use in our efforts to save the teeth? Is it the most compatible of all our list of filling materials with the dentine of the tooth, with the general tooth structure? And if so, is it the most compatible in all the cases that come under our care? Are we right in using it regardless of the character of the dentine, and enamel, with which it comes in contact? Who that has had any extensive experience would use gold in a cavity from decay in a chalky tooth, or in one attacked with white caries, and expect by this material to conserve that tooth, to check its disintegration, the continued breaking down of its substance for any length of time? In teeth, where the adjustment of the rubber dam is difficult, where there is a doubt of keeping the cavity absolutely dry throughout the operations, where the length of time consumed causes a tax on the patient's strength to such an extent, we feel compelled to hurry our work, thus failing to give it the care it requires to insure conservative and durable results. In approximal cavities in the posterior teeth, where the mouth is small and it is almost impossible to gain proper access, is gold the best thing to use? Would not some other material suggest itself as being more suitable to these conditions? Is not a perfect filling, made from a baser material, better than an imperfect one from the best? Why is it that we have so largely given up the use of tin in our approximal cavities that are accessible? It is certainly conservative in its character, and teeth have been saved by it for twenty or thirty years. Is it because we cannot obtain as remunerative a fee for tin as for gold, that we fail to use it? Is it that we are unwilling to have other members of the profession note our work, fearing adverse criticism, and possibly a suggestion that we were not equal to the emergency with the finer metal? Or is it that we doubt the saving qualities of that metal in the teeth? Experience justifies in saying, where tin does not have to bear the friction of attrition, where it will not be worn away by mastication, it is the equal if not the superior of gold as a conservative filling; and particularly is this the case in teeth of a soft structure.

A short time since an article appeared in one of our journals from a representative member of the dental profession, in which the statement was made that tin at the cervical wall of a cavity was of no value, and then he adds, "I have never used tin."

Are we to take such statements as this for a text on which to build our faith? Are we to be guided in our practice by the opinions of one person who acknowledges he has had no experience from which to form his conclusions?

Why do we use gold as a filling material? Is it because we find it saves the teeth better than anything else, or is it because it is soft in its character and conforms well to the shape of the cavity we wish to stop, and is pleasing to the eye, and is not glaringly in contrast to the color of the tooth, and being a pure metal does not discolor in the mouth, having no affinity for oxygen or sulphuretted hydrogen?

Basing an argument on the admission that gold is always preferable, the question arises, what kind shall we use?

To-day, many operators, capable of executing beautiful work, claim that cohesive gold is the only form by which perfect results may be obtained. Would it not be safe to suggest that those who make this claim have not learned to handle gold in any other way? They have had fair results from their methods; they have had failures. Do they attribute their failures to the character of the filling material, or to faulty manipulation, or to both? Pinning their faith to the one method they have been taught. Knowing no other, does it ever occur to them that some other material, or some other method of using the material with which they are familiar, might save them many annoying failures, and much mortification? Much antagonism is expected when the statement is made that soft or non-cohesive foil has more saving properties when introduced into a cavity in a tooth, than gold of a cohesive character. Antagonism is desired, that the statement may be thoroughly canvassed and disproved if found false.

The writer disclaims any intention or desire to inveigh against the use of cohesive foil. Its value is recognized; it has its place, and in some instances can accomplish that which cannot be done with foil in any other form; but the statement is ventured that it is less kindly received by the enamel and dentine, that it is less in harmony with the tooth structure, that in its use enamel margins are more liable to be broken down, and that it cannot be brought into as close contact with the walls of a cavity as foil that depends on other principles than cohesion for its retention. The use of non-cohesive foil does not depend on one method only. It may be used on the wedge principle, one part supporting another, each piece acting as the key-stone of an arch. It may be packed, on the principle of incorporation, one portion being locked, dovetailed, or incorporated into another mechanically, or it may be made to cohere by rotation of the instrument, for, while unlike so-called cohesive gold, non-cohesive gold is cohesive on the same principle that all pure metals are, by molecular cohesion, or attraction of their molecules. Foil that depends on the wedging, or other principles, aside from cohesion from heat, can be forced to the point desired, and pressure on its surface, whether from hand or mallet, is felt through its whole substance, and all additional pressure or blows serve to drive it more closely to its place. It spreads under the burnisher readily, and can be made sufficiently hard for all practical purposes, either in approximal or coronal surfaces—not as hard as cohesive foil, and it is just this hardness at the cervical walls that we wish to avoid, for undue density does not tend to conservatism. Cohesive foil is less tractable; it may be placed just where it is wanted, for it cannot be driven there. Pressure, instead of being felt through its substance, only serves to harden its surface, and all the blows that can be rained on the surface of a filling that is not in absolute contact with the walls of a cavity will not make it hug them more closely. In short, soft foil can be made to fit the cavity more thoroughly, to keep out moisture and microbes, and to prevent the recurrence of decay at the margins of the fillings, in the same way that a velvet cork will fit more closely, more securely, in the mouth of a bottle, than of a less yielding substance.

At the cervix of a cavity, and at all its margins, soft foil is preferable to cohesive, and in the approximal surfaces of the posterior teeth, tin or alloy is probably better than gold at the gingival margin where it can be used, (and it is seldom that it cannot) No pits or retaining points are necessary to start the filling, and these are always better dispensed with. In examining a mouth some time since, that had in it quite a number of large approximal fillings of cohesive foil, made by an expert operator, some ten or twelve years prior to the time it was seen, every one of the fillings were found defective at the cervical wall, secondary decay having occurred, excepting in one, the gingival margin having been filled with tin, on to which gold had been built. The marginal walls of this were perfect.

Is it easy to use non-cohesive foil skillfully? No! It is no lazy man's work. It requires strong muscular exertion. A filling (?) can be made of cohesive foil by the average operator that will remain in the cavity with more certainty than if made of soft foil, but the latter will be more conservative, and can be introduced in less than half the time, saving our own backs, and the neck of the patient from prolonged fatigue.

Will cohesive foil save teeth? Undoubtedly; but it requires a greater degree of patience, a larger expenditure of time, and more prolonged physical exhaustion than is required by the use of soft foil to produce equally good results.

Before the introduction of cohesive foil teeth were filled with gold that was non-cohesive, with soft gold, the softer the better; and these fillings, put in forty or more years ago, in many cases, are still standing, preserving the teeth, monuments of the skill of the workers of the material then almost exclusively used.

Have we deteriorated? Have we lost the skill, or rather failed to attain that of the old masters of fifty years ago, as the painter has failed to recover that of the old masters of his art of hundreds of years ago?

Why do fillings of gold fail? Sometimes from lack of judgment as to where it should be used, (for there are certain kinds of chalky teeth that gold will not save no matter how used or by whom,) and as frequently from a lack of skill how to use it.

There are certain fundamental rules that must not be neglected, that must ever be our guide when a tooth is filled with gold, if we wish success to attend our operations. One of these is to so manipulate the material that it will never, under pressure, draw away from the margins of the cavity. This can be accomplished by always having the filling higher in the centre than at the edges during the process of construction. If this is not done, a microscopic separation of gold from tooth is liable to follow, allowing fluids to enter, thus establishing secondary decay. This refers more particularly to approximal cavities in the posterior teeth, though it is a good rule to follow in every instance.

In the anterior teeth, in approximal surfaces, where the cavities are not slotted, the inner portion of the filling should always be in advance of the external, that there may not be left a pit at the cutting edge which is almost impossible to fill. That the cavity should be thoroughly dry is hardly necessary to mention, though, before the days of rubber dam, teeth have been successfully filled under water; but this was with non-cohesive foil in the form of cylinders. Many operators anneal their gold by passing it through the flame of a lamp. This is better avoided, as an excess of heat tends to make it harsh and less yielding under the instrument. Many other rules which should govern the operator in his manipulation of gold can only be learned by experience. Each one knows something of value which has escaped the observation of others, and a free ventilation of this subject will put all in possession of valuable information.

What of the alloys as conservers of dentine? They unquestionably have value, if used properly. What is the proper way to use them? Some state they should be used as dry as possible; others say too little mercury impairs their value. There is yet much to be learned about them. Undoubtedly much of the success and failure attending their use depends more in the manner of mixing than in manipulation. They do prevent decay at cervial walls, and in repairing extensive gold fillings they are better than gold itself. Before the days of alloy, or amalgam, as it was then called, teeth that were badly broken down were removed as there was no way known of saving them. To-day, comparatively useless shells are made serviceable for years by its use.

There seems to be a want of harmony between gold and certain portions of the calcific structure of the dental organs which is less noticeable in other metallic fillings. This is due either to the fluids of the oral cavity, (for it is that portion of the tooth around which the secretions linger that is most liable to give away) or to the imperfect adaptation of gold to marginal walls. Want of cleanliness enters largely into these conditions; but while this is a factor, it is not only cause, otherwise all metallic stoppings would produce a similar result. The ideal filling material is yet to be discovered.

The cements are valuable where they do not approach the cervix of the tooth, for at this point they disintegrate, or wash away rapidly. On the coronal surface they seem to waste only by attrition. We are all inclined to recommend and laud that with which we have had the greatest success. That is natural; but is liable to lead us into hobbies. An eclectic practice will make us more liberal in our views, and certainly tend to give our patients the best services.—*Southern Dental Journal.*

